# The association between interleukin‐1 polymorphisms and their protein expression in Chinese Han patients with breast cancer

**DOI:** 10.1002/mgg3.804

**Published:** 2019-07-11

**Authors:** Juan Wang, Yonggang Shi, Gaochao Wang, Shiliang Dong, Daoke Yang, Xiaoxiao Zuo

**Affiliations:** ^1^ Department of Radiation Oncology Zhengzhou University First Affiliated Hospital Zhengzhou Henan Province People's Republic of China; ^2^ Zhoukou Hospital of Traditional Chinese Medicine Zhoukou Henan Province People's Republic of China

**Keywords:** breast cancer, gene polymorphisms, IL‐1, IL‐1β, PCR‐RFLP, tumor markers

## Abstract

**Background:**

Breast cancer (BC) is the most common cancer in women and the second leading cause of cancer‐related deaths among women worldwide. Single nucleotide polymorphisms (SNPs) in cytokine genes have been shown to alter their expressions or functions in patients with BC. In recent years, the molecular structure and function of IL‐1 have been studied. Its genetic polymorphism could affect the transcription and expression of the IL‐1 gene. Moreover, it is closely related to several diseases. This fact and plethora of gene polymorphism data prompted us to investigate the relationship between IL‐1 polymorphisms and IL‐1 protein expression in Chinese Han BC patients.

**Method:**

In total, 298 patients with BC and 287 healthy control women were studied. The genetic polymorphisms for IL‐1 were analyzed by the MassARRAY sequencing method. Tumor markers and IL‐1β levels were measured by electrochemiluminescence and ELISA, respectively. All gene selection GRCh38 version.

**Results:**

The rs1143623 (NC_000002.12:g.112838252C>G) (GC), rs16944 (NC_000002.12:g.112837290A>G)(AG), and rs10490571 (NC_000002.12:g.102100877C>T) (CC) SNPs were found to be significantly lower in the BC group than in the controls. The variant G/C genotype of rs1143623 was associated with a significantly increased risk for BC (OR = 2.34, *p* < 0.05). The alleles for rs16944 (A/G; OR = 3.15, *p* < 0.05) and rs10490571 (T/C; OR = 2.48, *p* < 0.05) were also significantly associated with BC. Moreover, the genotypes of rs1143623, rs16944, and rs10490571 were significantly correlated with serum IL‐1β levels and other tumor markers.

**Conclusion:**

Our data reveal the association between genetic polymorphisms of IL‐1 and BC susceptibility in the Chinese Han population and indicates that IL‐1 polymorphisms are closely associated with tumor markers and IL‐1β protein expression in BC patients.

## 1 INTRODUCTION

Breast cancer (BC) is one of the most common cancers prevalent in women and is the second leading cause of cancer‐related deaths among women worldwide. According to Global Cancer Statistics data published in 2015, 1.7 million individuals were diagnosed with BC worldwide, and 521,900 of those died of the disease (Santtila, Savinainen, & Hurme, 1998). Although its exact etiology is still unclear, accumulating evidence suggests that BC is a multifactorial disease influenced by both genetic and environmental factors (Ferlay et al., 2012). Single nucleotide polymorphisms (SNPs) have been shown to alter their expressions or functions and play an important role in the onset and development of BC (Qian et al., 2010; Smyth, Cretney, Kershaw, & Hayakawa, 2004). The role of cytokines in cancer immunization and carcinogenesis has been recognized (Hirotsu et al., 2015).

The interleukin (IL)‐1 cytokine family is involved in inflammation and immune responses and includes IL‐1α, IL‐1β, and IL‐1 receptor antagonist (Ren et al., 2016).The human genes encoding *IL1A* (OMIM: 147760), *IL1B* (OMIM: 147720), and *IL1RN* (OMIM: 147679) are located within a 430 kb region on chromosome 2q14.2 (Xia, 2015). Polymorphisms of IL1A, IL1B, and IL1RN correspond with altered IL‐1α, IL‐1β, and IL‐1Ra protein expression in vitro and in vivo, respectively (Lacruz‐Guzmán et al., 2013; Zhou, 2015). The two cytokines, IL‐1α and IL‐1β, are important initiators and propagators of the inflammatory response. They are highly expressed in multiple cancers and are produced by tumor cells themselves, infiltrating myeloid cells and tumor‐associated fibroblasts (Dai et al., 2016). In cancer, IL‐1β has been more clearly established as a tumor promoting cytokine (Xia, Jin, et al., 2014). However, there is little information regarding the relationship between IL‐1 polymorphisms and its protein expression in patients with BC, particularly among the Chinese Han population.

Thus, based on the important roles of IL‐1 and IL‐1β in cancer and the previously reported associations of IL‐1 polymorphisms with various malignant diseases, we hypothesized that IL‐1 gene polymorphism may affect protein expression and play critical roles in BC pathogenesis in the Chinese Han population. Using polymerase chain reaction‐restriction fragment length polymorphism for genotyping, multiple methods for evaluating serum levels of IL‐1β and tumor markers, and powerful statistical methods, we examined the association of IL‐1 polymorphisms with BC.

## 2 MATERIALS AND METHODS

### 2.1 Study subjects

A total of 298 BC patients and 287 healthy controls were consecutively recruited between June 2012 and July 2016 at the First Affiliated Hospital of Zhengzhou University, People's Republic of China. The controls had no family history of BC and all had been clinically confirmed and/or had a recent mammogram confirming that there was no detectable BC at the time of sampling. Clinicopathological parameters including age, histological sub‐type, TNM stage, tumor grade, lymph node metastasis, age at menarche, menopause status, number of pregnancies, number of deliveries, and family history of cancer were evaluated. All recruited individuals signed an informed consent form and were asked to complete a self‐administered questionnaire.

### 2.2 Sample collection

About 5.0 ml of peripheral venous blood was collected in fasting state from the patients and control individuals for the estimation of biochemical parameters specific to BC, serum cytokine, and genotyping. Blood serum was freshly separated and stored in aliquots at −80°C before cytokine determination.

### 2.3 Diagnosis and tumor markers of BC

Clinical pathological results were used for diagnosis of BC. The tumor marker was detected by electrochemiluminescence analysis using the electrochemical luminometer (Roche cobase 411 of Roche Diagnostics Co., Ltd.) and Alpha fetoprotrin(AFP) and CAl25 reagents (Roche), according to manufacturer's instructions. The normal reference values for AFP, CA125 are <25 ng/ml, and <35 U/mL, respectively.

### 2.4 Cytokine assay

IL‐1β was quantified by standard ELISA kits (Omega Diagnostics Ltd., Alva, UK) using a microplate reader (Thermo Scientific, Hudson, NH, USA) as per manufacturer's protocol. All standards, BC samples, and control samples were run in duplicate.

### 2.5 SNP selection and genotyping

Three tag SNPs (rs1143623, rs16944, and rs10490571) in *IL1B* were selected for our study; these SNPs were minor allele frequencies > 5% in the HapMap Chinese Han Beijing population. Genomic DNA was isolated from whole‐blood samples using the GoldMag‐Mini Purification Kit (Gold Mag Co. Ltd. Xi'an, People's Republic of China) and DNA concentrations were measured using the NanoDrop 2000 (Thermo Scientific, Waltham, MA, USA). Sequenom MassARRAY Assay Design 3.0 software was used to design a multiplexed SNP Mass EXTENDED assay (Xia, Wang, et al., 2014). Genotyping was performed on a Sequenom MassARRAY RS1000 platform using the manufacturer's protocol. Data management and analysis were performed using the Sequenom Typer 4.0 Software (Bensen, Dawson, Mychaleckyj, & Bowden, 2001). All gene selection GRCh38 version.

### 2.6 Statistical analysis

Statistical analysis was carried out using SPSS software (version 25.0). Allele and genotype frequencies of IL‐1 polymorphisms were obtained by direct counts. SNP allele frequencies in the control subjects were tested for departure from Hardy–Weinberg equilibrium before analysis. Differences between the cases and controls in the distributions of demographic characteristics, selected variables, and allele frequencies of the three SNPs were evaluated using chi‐square tests for categorical variables and Welch's *t* tests for continuous variables. Data are expressed as mean ± standard deviation. Pearson's correlation analysis was done in BC subjects to determine the relation of tumor markers, serum IL‐1β levels, and its genetic variants. The comparison of prevalence of different genotypes in different subject groups, the odds ratios, and 95% confidence interval were calculated using logistic regression analysis. *p* values <5% were considered significant.

## 3 RESULTS

### 3.1 Demographic profile and biochemical parameters of study subjects

A total of 298 patients with BC and 287 healthy individuals were enrolled in the study. Basic characteristics of the patient and control groups are described in Table 1. There were no significant differences in the distributions of age and menopausal status between BC patients and healthy controls (*p* > 0.05). The proportion of patients with higher clinical stages (II/III/IV) was 56.4%. The percentages of patients with positive estrogen receptor, positive progesterone receptor, and negative human epidermal growth factor receptor 2 were 58.4%, 47.7%, and 39.3%, respectively.

**Table 1:** Basic characteristics of patients with breast cancer and of the control individuals

Characteristics	Cases (*N* = 298)	Controls (*N* = 287)	*p*‐Value
Age			0.304^a^
Mean ± *SD*	49.54 ± 10.52	50.34 ± 8.13	
Menopause			0.900^b^
Premenopausal	121 (40.6%)	118 (41.1%)	
Postmenopausal	177 (59.4%)	169 (58.9%)	
Clinical Stages			
I	130 (43.6%)		
II/III/IV	168 (56.4%)		
Estrogen Receptor			
Negative	124 (41.6%)		
Positive	174 (58.4%)		
Progesterone Receptor			
Negative	156 (52.3%)		
Positive	142 (47.7%)		
HER2			
Negative	117 (39.3%)		
Positive	181 (60.7%)		

Abbreviations: HER2, human epidermal growth factor receptor 2; *SD*, Standard deviation.

a *p* value was calculated by Welch's *t* test.

b *p* value was calculated by Pearson's χ2 test.

### 3.2 Serum tumor markers

The serum tumor marker levels in the subjects are presented in Table 2. Significantly higher levels of CA125 and AFP were observed in BC patients compared with those in the control group, which confirmed the positive test for patient selection. Serum levels of IL‐1β and IL‐1R1 were also significantly higher in BC patients in comparison to controls (*p* < 0.05).

**Table 2:** Cytokine and inflammatory markers among control and BC patients

	Control	BC patients
IL‐1β	122.34 ± 1,011.34	6.24 ± 3.86^a^
IL‐1R1	0.16 ± 0.10	0.14 ± 0.08^a^
CA125	32.97 ± 36.00	41.97 ± 64.22^a^
AFP	13.71 ± 3.53	29.28 ± 120.48^a^

Note

Data are presented as mean ± *SD*. Comparison between the groups was performed with one‐way ANOVA.

Abbreviations: AFP, alpha fetoprotein; BC, breast cancer; CA125, cancer antigen.

a *p* < .05, control versus RA patients.

### 3.3 Gene polymorphism (rs1143623, rs16944, and rs10490571)

The genotype and allele frequency distributions of SNPs rs1143623, rs16944, and rs10490571 are presented in Table 3. The genotypes for rs1143623(GC), rs16944(AG), rs10490571(CC) were found to be significantly lower in the BC group as compared with those in the control group. To evaluate the association at the genotype level, the homozygous mutant group was combined with the heterozygous group, and logistic regression analysis was carried out. For rs1143623, the heterozygous mutant variant (G/C) was significantly associated with BC patients, and not with the controls (OR = 2.34, *p* < 0.05). The homozygous mutant variant (GG) of the same allele was also significantly associated with BC patients, and not with the controls (OR = 3.51, *p* < 0.05). Additionally, the association persisted when we combined the heterozygous and homozygous mutants (GC + GG), when compared with controls (OR = 2.29; *p* < 0.05).

**Table 3:** Allelic and genotypic frequency of rs1143623(G/C), rs16944(A/G), and rs10490571(T/C) polymorphism among control and BC patients

	Control (*N* = 287)	BC patients (*N* = 298)
rs1143623		
GG	36 (12.5%)	54 (18.1%)
GC	160 (12.5%)	132 (44.3%)
CC	91 (31.7%)	112 (37.6%)
G allele	118 (41.1%)	123 (41.3%)
C allele	169 (58.9%)	175 (58.7%)
rs16944		
AA	56 (19.5%)	76 (25.5%)
AG	163 (56.8%)	132 (44.3%)
GG	68 (23.7%)	90 (30.2%)
A allele	137 (47.7%)	141 (47.3%)
G allele	150 (52.2%)	156 (52.3%)
rs10490571		
TT	7 (2.4%)	14 (4.7%)
TC	87 (30.3%)	96 (32.2%)
CC	193 (67.2%)	188 (63.1%)
T allele	43 (15.0%)	67 (22.5%)
C allele	244 (85.0%)	231 (77.5%)

Abbreviation: BC, breast cancer.

Similarly, the rs16944 heterozygous mutant variant (A/G) and rs10490571 heterozygous mutant variant (T/C) were significantly associated with BC patients, and not with those in the control group (OR = 3.15 and OR = 2.48, respectively; *p* < 0.05). The homozygous mutant variants of rs16944(AA) and rs10490571(TT) were also significantly associated with BC patients, and not with control individuals (OR = 2.67 and OR = 1.73, respectively; *p* < 0.05). Furthermore, the association for both alleles persisted when we combined the heterozygous and homozygous mutants (AA + AG and TC + TT), when compared with controls (OR = 3.42 and OR = 2.74; *p* < 0.05 and *p* < 0.05, respectively) (Table 4).

**Table 4:** Odds ratio for presence of different genotypes in rs1143623, rs16944, and rs10490571 polymorphism between control and BC patients

Genotype	Odds ratio	95% confidence interval	*p*‐Value
rs1143623			
GC vs. CC	2.3376	1.4558–3.4163	0.0012
GG vs. CC	3.5141	1.54300–6.5804	0.0013
GG + GC vs. CC	2.2933	1.6104–3.5775	0.0020
rs16944			
AG vs. GG	3.1452	1.6573–4.4274	0.0013
AA vs. GG	2.6736	1.7643–7.2516	0.0002
AA + AG vs. GG	3.4159	1.4509–3.2578	0.0016
rs10490571			
TC vs. CC	2.4832	2.0623–3.0742	0.0023
TT vs. CC	1.7348	1.5616–1.9687	0.0026
TT + TC vs. CC	2.7366	2.2245–3.4681	0.0020

Abbreviation: BC, breast cancer.

### 3.4 Association of tumor markers with IL1B polymorphism among BC patients

Association of tumor markers with *IL1B* polymorphism among BC patients is presented in Table 5. The genotypes of rs1143623, rs16944, and rs10490571 were significantly correlated with IL‐1β, CA125, and AFP levels in the serum. For rs1143623, the differences in IL‐1β level between GG and CC, or GC and CC were statistically significant. The differences in CA125 and AFP levels between GG and GC, or GC and CC were statistically significant. Similarly, for rs16944, the differences in IL‐1β and CA125 levels were statistically significant between AA and AG, AG and GG, or AA and GG, while the differences in AFP were statistically significant between AA and AG or AG and GG. In case of rs10490571 allele, the differences in IL‐1β levels between TT and TC, or TC and CC were statistically significant. The differences in CA125 levels between TT and TC, TC and CC, or TT and CC were statistically significant. The differences in AFP levels between TC and CC, or TT and CC were statistically significant.

**Table 5:** Association of different genotypes with serum levels of IL‐1β, CA125, and AFP among BC patients^a^

	Genotypes	*N*	IL−1β	CA125	AFP
Rs1143623	GG	54	10.17±7.45^b^	24.76±5.99^b^	9.32±3.82^b^
	GC	132	8.92±4.85^b^	44.60±8.44^c^	35.48±6.40^c^
	CC	112	3.53±3.20^c^	25.99±4.18^b^	10.53±4.29^b^
rs16944	AA	76	12.23±10.96^b^	33.09±8.09^b^	8.52±3.08^a^
	AG	132	7.30±3.21^c^	37.93±7.74^c^	51.38±20.27^c^
	GG	90	8.80±4.44^d^	28.81±10.65^d^	9.63±4.00^b^
rs10490571	TT	14	0.14±0.02^b^	41.46±8.76^b^	13.90±2.25^b^
	TC	96	0.98±0.59^c^	35.55±7.02^c^	13.94±3.50^b^
	CC	188	0.17±0.82^b^	32.65±10.24^d^	29.39±6.74^c^

Note

a, b and c represent the same letters in pairwise comparison with statistical significance, While different letters have no statistical significance.

a Data are presented as mean ± *SD*, one‐way ANOVA followed by Post Hoc Tukey's test.

## 4 DISCUSSION

In this study, we investigated the associations between IL‐1 polymorphisms and its protein expression in Chinese Han patients with BC. We found that rs1143623, rs16944, and rs10490571 SNPs are significantly associated with BC risk. The genotypes of rs1143623, rs16944, and rs10490571 were significantly correlated with serum IL‐1β, CA125, and AFP levels. Moreover, our results indicate that the polymorphisms of IL‐1 affected the protein expression of serum IL‐1β, CA125, and AFP in patients with BC in the Chinese Han population.

IL‐1 is a large family of cytokines, which could regulate innate immune responses to protect the host from pathogens (Nicklin, Weith, & Duff, 1994). IL‐1 has been shown to inhibit growth of BC cells and to promote cellular differentiation in vitro. It is also known to stimulate the expression of several proteolytic enzymes in human cancer (Tarlow et al., 1993). IL‐1β was found to be elevated in various types of cancers, and IL‐1β producing tumors have been associated with bad prognosis (Danis, Millington, Hyland, & Grennan, 1995). IL‐1β is a pleiotropic cytokine implicated in tumor progression through effects on proliferation, invasion, metastases, myeloid cell recruitment, and angiogenesis (Hurme & Santtila, 1998). It has been reported that IL‐1β expression in BC is associated with receptor‐negative disease, macrophage infiltration, and poor outcome (di Giovine, Takhsh, Blakemore, & Duff, 1992).

However, until now, no studies have reported the association of IL‐1β expression in BC with interleukin‐1 gene polymorphism. Our study is the first to demonstrate that polymorphisms in *IL‐1* is associated with serum IL‐1β, CA‐125, and AFP expression and that it affects IL‐1 expression in patients with BC in the Chinese Han population. Although this study had sufficient statistical power, there were still some intrinsic limitations.

## CONFLICT OF INTEREST

The authors declare that there is no conflict of interest with this manuscript.
